# Explant analysis of a chromium nitride coated metal-on-metal total wrist replacement: A case study

**DOI:** 10.1177/09544119241277697

**Published:** 2024-09-10

**Authors:** Thomas J Joyce, Göksu Kandemir, Duncan McGuire, Michael Solomons, Daniel J Brown

**Affiliations:** 1 School of Engineering, Newcastle University, Newcastle upon Tyne, UK; 2 Martin Singer Hand Unit, Groote Schuur Hospital, Cape Town, South Africa; 3 Royal Liverpool University Hospital, Liverpool, UK; 4 Faculty of Health and Life Science, University of Liverpool, Liverpool, UK

**Keywords:** Total wrist replacement, explant analysis, roughness, chromium nitride coating, articulating surfaces, cobalt chromium, metal-on-metal

## Abstract

Explant analysis can provide important understanding of how artificial joints perform in the human body. The articulating surfaces of the metacarpal head and the radius cup from a chromium nitride coated metal-on-metal Motec wrist implant were analysed. Due to bone resorption and aseptic loosening, the implant was removed after 6 years in the patient, and metallosis was observed during removal. Visually, some areas of the articulating surfaces appeared polished, others were dulled. A chemical composition analysis of the metacarpal head showed that the polished surfaces were chromium rich, implying this surface was the original chromium nitride coating, whereas the dulled surfaces were cobalt rich, indicating the underlying cobalt chromium substrate. In addition, the underlying cobalt chromium substrate was an order of magnitude rougher than the polished surface, indicating the scale of damage to it. It is speculated that the loss of the coating, and the subsequent damage to the underlying substrate due to a third-body wear process, led to osteolysis and the metallosis seen at revision surgery.

## Introduction

**Figure 1.**
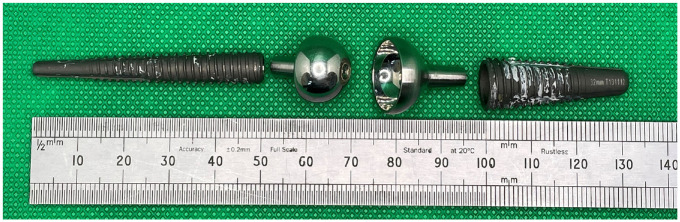
An example of an explanted metal-on-metal version of the Motec wrist prosthesis. From left to right, the titanium alloy metacarpal screw, the CoCr metacarpal head, the CoCr radius cup, the titanium alloy radius screw.

Total wrist arthroplasty (TWA) aims to restore pain-free motion to diseased wrist joints.^1^ For many years, and still often the case today, most TWAs employed a metal-on-polymer bearing combination, similar to that seen in the more commonly implanted hip and knee joint replacements.^2^ However, TWA has not been as successful as hip and knee arthroplasty.^3^ This lack of success was one of the reasons for the introduction of a new design of TWA, the Motec (Swemac Innovations AB, Linköping, Sweden), which offered different bearing combinations. The most common Motec bearing combination is a metal-on-metal (MoM) articulation,^4^ while a metal-on-carbon fibre reinforced polyether ether ketone (CFR-PEEK) is also available.^5^ In both cases the metal is Cobalt Chromium (CoCr) alloy, similar to that used in millions of artificial joints. All MoM Motec TWAs consist of a CoCr metacarpal head and a CoCr radius cup, and most commonly these have a nominal 15 mm articulating diameter. Each of these articulating components is held within its own titanium alloy ‘threaded screw’. An explanted metal-on-metal Motec TWA is shown in Figure 1, to indicate the metacarpal head, radius cup and the two threaded screws.

Historically, Swemac also offered a version of the Motec MoM TWA which had the CoCr bearing surfaces coated with Chromium Nitride (CrN).^6^ The aim of such a coating is to reduce wear.^7^ For context, the performance of ‘low wear’ coatings applied to joint replacements has not always been successful, with catastrophic failures of diamond like carbon^8,9^ and titanium niobium^10^ coatings reported. In the latter case, ‘massive osteolysis’ leading to aseptic loosening was seen clinically.^11^ However, for CrN coatings, encouraging in vitro results have been offered.^12^ To investigate this area further, for the first time, an explanted CrN coated MoM Motec TWA was analysed.

## Materials and methods

### The explanted CrN coated Motec TWA

**Figure 2.**
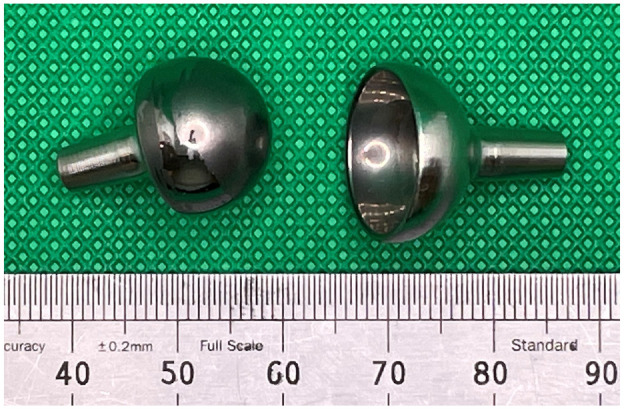
Image showing the articulating surfaces of the explanted CrN coated MoM Motec metacarpal head (left) and the radius cup (right).

The articulating surfaces of an explanted 15 mm diameter CrN coated MoM Motec TWA (Figure 2) were analysed. The titanium threaded screws were not available for analysis, as these had been left in situ when the CrN coated components were replaced with a CoCr on CFR-PEEK articulation.

### Articulating surface analysis

A Zygo NewView 5000 non-contacting profilometer (with a sensitivity of 0.001 µm, Sa^13^) was used to analyse the articulating surfaces of the metacarpal head and radius cup of the explanted Motec TWA. Eight roughness measurements were taken from each articulating surface. For the metacarpal head, measurements were taken from its entire surface. However, due to the physical size of the Zygo lens, measurements from the radius cup were only taken from its polar region.

An elemental analysis on the articulating surface of the explanted metacarpal head was undertaken using a Thermo Fisher Niton XL3t XRF (X-Ray Fluorescence) Analyzer. With this non-destructive technique, information on the chemical composition of materials can be obtained. Five measurements were taken from the pole and equator of the explanted head, and the percentage compositions were averaged. Unfortunately, XRF measurements could not be undertaken on the articulating surface of the explanted radius cup due to geometric limitations.

### Clinical details

**Figure 3.**
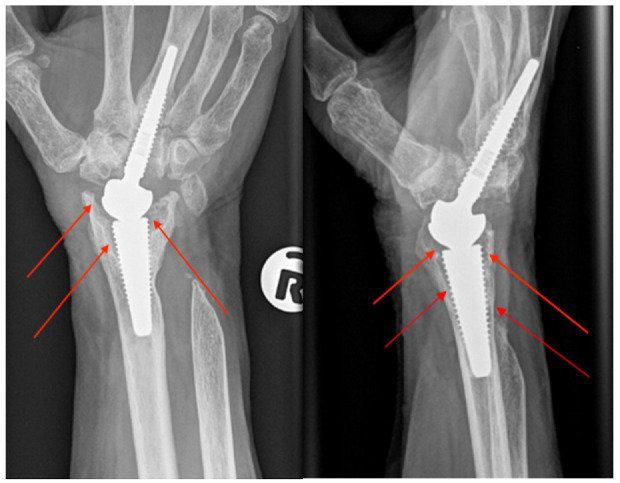
Posteroanterior and lateral X-ray image of the implanted CrN coated MoM Motec TWA. The red arrows show the bone resorption observed around the radius threaded screw which is seen as a progressive black line surrounding the screw, often more noticeable adjacent to the joint.

A female patient who was diagnosed with rheumatoid arthritis (aged 68 at implantation) was treated with a medium-neck CrN coated MoM Motec TWA in 2012. The patient was sedentary and did not undertake any sport or other activities that would excessively load the wrist. Approximately 3 years after the primary operation, bone resorption was first observed on X-rays around the radius threaded screw, and aseptic loosening was recorded (Figure 3). Due to progressive osteolysis and synovitis, revision surgery was undertaken 6 years after the initial implantation.

**Figure 4.**
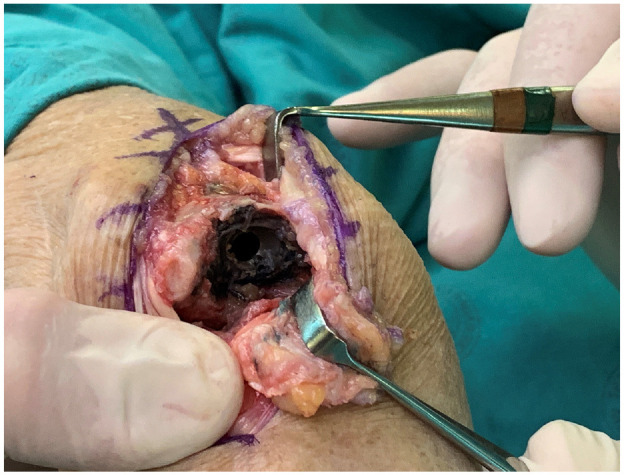
Metallosis was seen at removal of the CrN coated MoM Motec TWA.

Metallosis (black staining of tissue due to metal debris)^14^ was observed around the implant during removal (Figure 4).

At the time of revision surgery, the screws were found to be stable, despite the radiographic lysis, and were therefore retained. The articulation was changed to metal on CRF-PEEK. The revision surgery was successful and at the last follow up, 5 years following the revision, the patient had good function and no pain with functional range of motion as follows: flexion 10°, extension 50°, pronation 80°, supination 80°.

## Results

### Explant visual analysis

**Figure 5.**
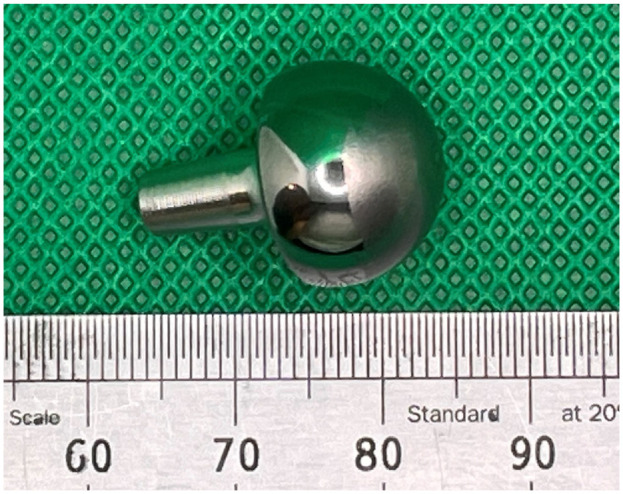
The explanted CrN coated CoCr metacarpal head. A polished region (left hand side of the head) and a dulled region (right-hand side of the head) can be seen. Scale in mm.

Two distinctive regions could be seen on the articulating surface of each component; one region had a polished appearance and the other had a dull appearance. On the explanted metacarpal head, the dull regions were mainly concentrated around an expanded polar region but there were also dull regions in the shape of wide stripes around the equator (Figure 5). The remainder of the articulating surface, including a small region at the pole, appeared polished.

**Figure 6.**
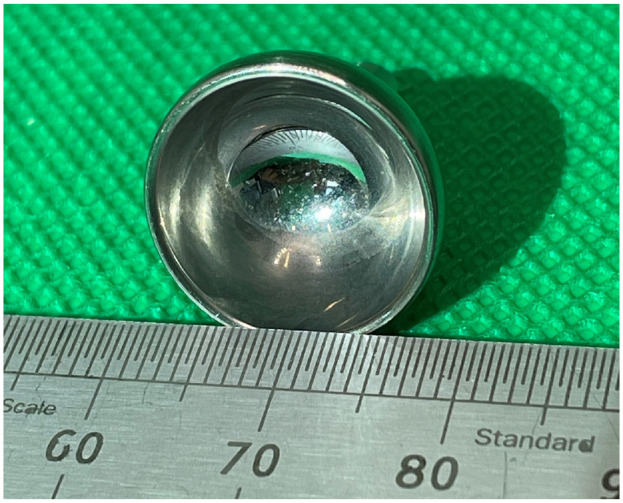
The explanted CrN coated CoCr radius cup. Note the polished polar region surrounded by a dulled equatorial region. Scale in mm.

For the explanted radius cup, the dull regions could be most clearly seen at the equator and extending towards the pole, as shown in Figure 6. In contrast, the polar region appeared polished.

### Roughness analysis

For the metacarpal head, mean roughness values measured were 0.704 ± 0.209 μm (Sa) for the dull region and 0.058 ± 0.021 μm (Sa) for the polished region. For the radius cup, only the roughness of the polished polar region could be measured, and it had a roughness of 0.073 ± 0.035 μm (Sa). The average roughness of the polished regions observed on the metacarpal head and the radius cup were not found to be significantly different (*p*: 0.36, *t*-test).

### XRF results

Table 1 shows the average of the five measurements taken using the XRF Analyser from the pole (dull region) and the equator (polished region) of the explanted metacarpal head. The preponderance of Cr at the polished equator, indicating CrN, should be clear; so too the preponderance of Co at the dull pole indicating the CoCr substrate.

**Table 1.** Material chemical composition (mass fraction in percentage %) of the explanted metacarpal head measured at its pole (the dull region) and equator (polished region) using an XRF analyzer.

Material	Cr	Mn	Fe	Co	Ni	Mo
Pole	38.58 ± 3.01	0.33 ± 0.05	0.17 ± 0.04	55.84 ± 2.79	0.15 ± 0.09	4.75 ± 0.12
Equator	58.16 ± 6.44	0.06 ± 0.15	0.02 ± 0.05	36.85 ± 6.05	0.48 ± 0.08	4.30 ± 0.38

### Localised changes to the CrN coating

**Figure 7.**
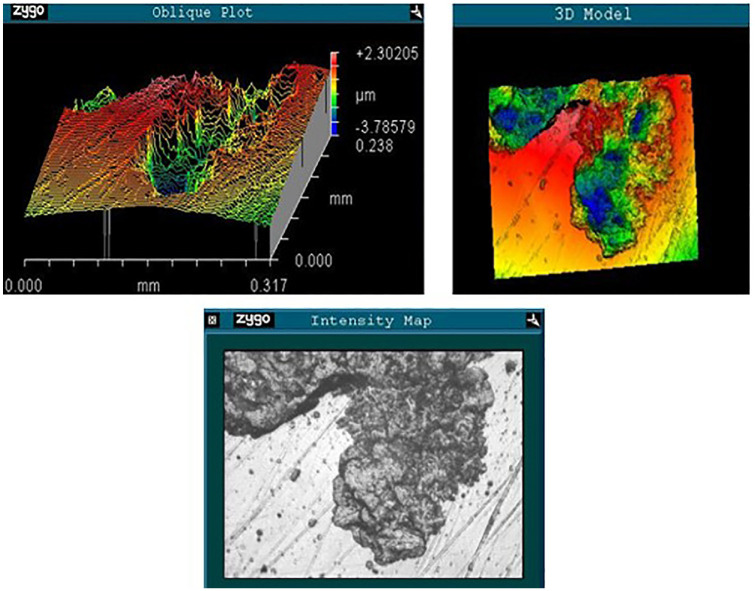
Zygo images taken from the intersection of coating and removed coating near the equator of the explanted metacarpal head (Sa: 0.907 μm). Top left is the Zygo oblique plot, top right is the Zygo 3D model, and bottom is the intensity map showing the polished and dull regions.

A composite set of images taken from the Zygo non-contacting profilometer, Figure 7, shows the localised topography at a point near the equator on the metacarpal head where the coating and the worn region met. The localised loss of coating should be clear. The retained coating appears as a smooth curved surface on the oblique plot (top left), with the area of coating removal appearing as a relatively deep valley.

## Discussion

This is the first study to report on an explanted CrN coated MoM Motec TWA. It should be noted that this describes a historical issue as the CrN coating was introduced in 2009 and withdrawn in 2012 as it was felt that the coated implant would not be beneficial with the new CRF-PEEK articulation.

As shown by the XRF data, the polished region at the equator of the metacarpal head had a preponderance of Cr, thus indicating that the polished region was the original CrN coating. In contrast, the XRF data for the dull region at the pole of the metacarpal head showed a preponderance of CoCr, and thus the substrate material of the MoM TWA. As further evidence of this ‘polished = CrN’ and ‘dull = CoCr’, the average roughness measurements of the polished region of the metacarpal head were 0.058 μm, while that of the dull region, was over an order of magnitude greater at an average roughness of 0.704 μm. The polished surface of the explanted cup had an average roughness of 0.073 μm. This value was not found to be statistically different to the coated region of the explanted head (*p* > 0.05). At an average roughness of 0.704 μm, this is far above the maximum value of 0.050 μm Ra recommended for a metallic articulating surface of a joint replacement.^15^ Therefore, the magnitude of damage to the underlying CoCr surface should be clear.

Much of the original CrN coating of the articulating surfaces had been removed. It was likely this relatively hard debris which served as third body wear particles to cause wear of the CoCr substrate. In turn, the combination of CrN and CoCr particles are the likely cause of the metallosis seen at revision surgery (Figure 5). A similar phenomenon has been reported for a diamond like carbon coated MoM metatarsophalangeal joint replacement,^8,9^ and for a titanium niobium coated MoM proximal interphalangeal joint replacement.^10^

In the case of the diamond like carbon coated MoM metatarsophalangeal joint replacement,^8,9^ it was removed approximately 4 years after implantation. At revision, metallosis and osteolysis were reported,^8^ the same as with the CrN coated Motec. The diamond like carbon coating had been removed from all of the articulating surface of the part-spherical, concave CoCr phalangeal component, and most of the articulating surface of the part-spherical, convex CoCr metatarsal component.^8^ The extensive nature of the coating removal is similar to that seen with the CrN coated Motec. Hopefully then the link between extensive coating removal and damage to the CoCr substrate, and metallosis and osteolysis is credible. In another study, of five components of a titanium niobium coated MoM proximal interphalangeal joint replacement, again substantial loss of coating material was seen.^10^ In a review of all the titanium niobium coated MoM proximal interphalangeal joint replacements implanted in the UK, ‘massive osteolysis’ and metallosis were reported, at a maximum follow-up of 6 years.^11^ Again, the link between loss of coating and damage to the CoCr substate seen in explanted components, in turn linked to osteolysis and metallosis reported clinically, should hopefully be clear.

What might have caused failure of the CrN coating? For the radius cup (Figure 6) a wide and dulled equatorial region is seen. This could suggest equatorial binding, as has been reported for some of the early metal-on-metal hips.^16,17^ However, this hypothesis is not supported by the appearance of the articulating surface of the metacarpal head; if equatorial binding took place, then the pole should be shiny, but it is not, instead it is dull. For a diamond like carbon coating applied to a metatarsophalangeal joint replacement, failure was shown to be due to poor corrosion resistance of the interface layer between the coating and the substrate.^9^ More work is required on the failure of the CrN coating and will be the basis of future research.

It is possible that, in part, implant failure may have been due to impingement related osteolysis.^18^ As noted, the titanium screws were left in situ and so these were unavailable for analysis, however there was no evidence of notching of the screw on the lateral radiograph (Figure 3) as seen in the published series.^18^ Further, the substantial removal of the CrN coating seen on both the metacarpal head and the radius cup strongly suggests that the metal debris arose from the articulating surfaces, not the titanium screws. Moreover, the XRF analysis (Table 1) showed no titanium. This is in contrast to impingement related osteolysis, where titanium was identified on the bearing surface of a short-neck MoM Motec TWA.^18^ In addition, the CrN Motec TWA was a medium neck, and we have not seen impingement related osteolysis with other medium neck Motec TWAs.^4,5^

The high values of roughness (average 0.704 µm Sa) measured on the CoCr surface of the metacarpal head contrast with those measured in a cohort of seven explanted MoM Motec TWAs (average 0.028 µm Sa)^4^ and seven explanted metal-on-CFR-PEEK Motec TWAs (average 0.019 µm Sa).^5^ This contrast in roughness values implies that there is a different failure mode with the CrN Motec TWA, than with uncoated Motec TWAs.

This study has some limitations. One was the sample size; only one CrN MoM Motec TWA was available for analysis. Whether this is an isolated and unrepresentative failure or whether it instead represents the first of a common mode of failure for this coating is not known at this stage. Our experience with other medical implants such as metal-on-metal hips^19^ and spinal growing rods,^20^ would suggest it is the latter. Moreover, had the CrN explant have shown low surface roughness, an intact CrN coating and was not associated with metallosis, then we may have taken a different view. We note that this study is the first to report an assessment of an explanted, CrN coated, MoM TWA and we would suggest that even a single sample explant analysis can be useful in terms of understanding the in vivo performances of artificial joints, as has been shown previously.^8,21–24^ Among this understanding are positive assessments of explants, such as the minimal surfaces changes seen on a Maestro wrist after 8 years in vivo.^22^

Another limitation was the small size of the components which caused some geometrical limitations, especially for the analysis of the explanted radius cup. Most of the roughness measurements and the XRF readings could only be taken from the explanted metacarpal head. The results obtained from the explanted head, however, gave insights into the changes that occurred in vivo on its counterface.

A further limitation is that no ion concentrations were measured pre- or post-revision surgery. However, the patient had good function and no pain after the revision and at the last follow up, which was 5 years following the revision. In addition, there were no kidney problems.

Surgeons who identify osteolysis around implants inserted at that time should confirm via the manufacturer/distributor if the implant had a ceramic coating, as if so, a simple exchange of the bearing surfaces would likely stop the osteolysis progressing.

Might the CrN coating issue be only a hand-wrist specific problem? Our view would be to take a precautionary approach and to suggest it could apply in all joints replaced in the human body. Moreover, as the loading in the wrist is substantially lower than in the lower limb, or the proximal joints of the upper limb, based on this case study we would caution against usage of CrN coatings. Failures in artificial joints can have a major effect on the lives of patients, and therefore need to be understood and eliminated wherever possible^25^

The novelty of this work is that, for the first time an explanted CrN coated Motec wrist joint replacement has been analysed. While CrN coatings on CoCr joint replacements were claimed to show low wear based on hip simulator studies,^12^ our case study based on implantation in a human subject calls such claims into question. Rather than this questioning be used to criticise simulators and in vitro testing, we would rather ask for such scepticism to be applied to the claimed benefits of ‘low-wear’ coatings for the articulating surfaces of joint replacements. Patients need to be protected from such untested implants and if the impact of our work is to achieve a note of caution, then we will have succeeded.

Overall, while the exact cause of failure of the CrN coating has not been determined, its failure has been shown and is the likely explanation for the osteolysis and metallosis seen clinically. The result also adds to the general caution that should be applied to ‘low wear’ coatings in joint replacements; any failure of the coating is likely to be catastrophic in terms of the relatively high wear volumes of metallic debris generated.

## Conclusion

An explanted CrN coated MoM Motec TWA was analysed. The CrN coating had been removed from substantial areas of the articulating surfaces, and this loss was likely the primary reason for metallosis and osteolysis seen clinically, and thus the failure of the artificial joint. This damage has not been seen in any other non-CrN coated Motec explants with any combination of bearing surfaces.
